# Supplementary material for: The Mitochondrial Genomes of Two Parasitoid Wasps Protapanteles immunis and Parapanteles hyposidrae (Hymenoptera: Braconidae) with Phylogenetic Implications and Novel Gene Rearrangements
Source: Genes (Basel). 2023 Jan 16;14(1):230. doi: 10.3390/genes14010230 (PMC9859417; doi:10.3390/genes14010230)
Supplement: Supplementary file 1 [file genes-14-00230-s001.zip › genes-2138378-supplementary.pdf]

Supplementary Materials

Table S1. Codon usage in the mitochondrial genome of *Pa. hypsidrae*.

Amino acid	Codon	Number	RSCU	Amino acid	Codon	Number	RSCU
Ala	GCA	26	1.6508	Pro	CCA	47	1.9583
	GCC	1	0.0635		CCC	1	0.0417
	GCG	2	0.1270		CCG	0	0
	GCU	34	2.1587		CCU	48	2
Cys	UGC	0	0	Gln	CAA	39	1.9024
	UGU	29	2		CAG	2	0.0976
Asp	GAC	1	0.0328	Arg	CGA	19	1.7674
	GAU	60	1.9672		CGC	0	0
Glu	GAA	70	1.9718		CGG	0	0
	GAG	1	0.0282		CGU	24	2.2326
Phe	UUC	12	0.0570	Ser	AGA	72	1.8403
	UUU	409	1.9430		AGC	0	0
Gly	GGA	98	2.5290		AGG	3	0.0767
	GGC	0	0		AGU	32	0.8179
	GGG	3	0.0774		UCA	126	3.2204
	GGU	54	1.3935		UCC	3	0.0767
His	CAC	1	0.0308		UCG	1	0.0256
	CAU	64	1.9692		UCU	76	1.9425
Ile	AUC	5	0.0200	Thr	ACA	52	2.1224
	AUU	496	1.9800		ACC	2	0.0816
Lys	AAA	147	1.9470		ACG	0	0
	AAG	4	0.0530		ACU	44	1.7959
Leu	CUA	6	0.0576	Val	GUA	40	1.5686
	CUC	0	0		GUC	0	0
	CUG	0	0		GUG	2	0.0784
	CUU	8	0.0768		GUU	60	2.3529
	UUA	597	5.7312	Trp	UGA	78	1.9259
	UUG	14	0.1344		UGG	3	0.0741
Met	AUA	328	1.8960	Tyr	UAC	8	0.0762
	AUG	18	0.1040		UAU	202	1.9238
Asn	AAC	7	0.0502				
	AAU	272	1.9498				

Table S2. Codon usage in the mitochondrial genome of *Pr. immunis*.

Amino acid	Codon	AA	Number	RSCU	Amino acid	Codon	AA	Number	RSCU
Ala	GCA	A	16	1.0667	Pro	CCA	P	32	1.3617
	GCC	A	0	0		CCC	P	1	0.0426
	GCG	A	0	0		CCG	P	2	0.0851
	GCU	A	44	2.9333		CCU	P	59	2.5106
Cys	UGC	C	0	0	Gln	CAA	Q	37	1.8974
	UGU	C	35	2		CAG	Q	2	0.1026
Asp	GAC	D	0	0	Arg	CGA	R	21	1.9535
	GAU	D	61	2		CGC	R	1	0.0930
Glu	GAA	E	67	2		CGG	R	0	0
	GAG	E	0	0		CGU	R	21	1.9535
Phe	UUC	F	3	0.0134	Ser	AGA	S	71	1.9254
	UUU	F	446	1.9866		AGC	S	0	0
Gly	GGA	G	80	2.2222		AGG	S	2	0.0542
	GGC	G	1	0.0278		AGU	S	24	0.6508
	GGG	G	1	0.0278		UCA	S	116	3.1458
	GGU	G	62	1.7222		UCC	S	3	0.0814
His	CAC	H	1	0.0323		UCG	S	0	0
	CAU	H	61	1.9677		UCU	S	79	2.1424
Ile	AUC	I	6	0.0267	Thr	ACA	T	62	2.4314
	AUU	I	444	1.9733		ACC	T	2	0.0784
Lys	AAA	K	151	1.9484		ACG	T	0	0
	AAG	K	4	0.0516		ACU	T	38	1.4902
Leu	CUA	L	3	0.0306	Val	GUA	V	42	1.4359
	CUC	L	0	0		GUC	V	0	0
	CUG	L	0	0		GUG	V	2	0.0684
	CUU	L	8	0.0816		GUU	V	73	2.4957
	UUA	L	564	5.7551	Trp	UGA	W	76	1.9740
	UUG	L	13	0.1327		UGG	W	1	0.0260
Met	AUA	M	344	1.9380	Tyr	UAC	Y	1	0.0101
	AUG	M	11	0.0620		UAU	Y	198	1.9899
Asn	AAC	N	1	0.0070					
	AAU	N	285	1.9930					

Table S3. Information of mitochondrial genomes used in phylogenetic analysis.

Family	Subamily	Tribe	Species	Accession number
Braconidae	Microgastrinae	Apantelini	*Choeras grammatitergitus*	KT215850
Braconidae	Microgastrinae	Apantelini	*Dolichogenidea* sp.	KT215851
Braconidae	Microgastrinae	Cotesiini	*Protapanteles* sp.	KT215853
Braconidae	Microgastrinae	Cotesiini	*Diolcogaster* sp.	KT215854
Braconidae	Microgastrinae	Cotesiini	*Cotesia vestalis*	NC_014272
Braconidae	Microgastrinae	Cotesiini	*Cotesia flavipes*	NC_063945
Braconidae	Microgastrinae	Microgastrini	*Microgaster campestris*	KT215844
Braconidae	Microgastrinae	Microgastrini	*Hygroplitis sinica*	KT215856
Braconidae	Microgastrinae	Microgastrini	*Microgaster* sp.	KT215857
Braconidae	Microgastrinae	Microplitini	*Microplitis incurvatus*	KT215845
Braconidae	Microgastrinae	Microplitini	*Snellenius* sp.	KT215848
Braconidae	Microgastrinae	Microplitini	*Snellenius radicalis*	KT215849
**Outgroup**				
Braconidae	Macrocentrinae		*Macrocentrus camphoraphilus*	GU097656
Braconidae	Agathidinae		*Therophilus festivus*	KF385868
